# Economical Value of Vaccines for the Developing Countries—The Case of Instituto Butantan, a Public Institution in Brazil

**DOI:** 10.1371/journal.pntd.0001300

**Published:** 2011-11-29

**Authors:** Paulo Lee Ho, Eliane Namie Miyaji, Maria Leonor Sarno Oliveira, Waldely de Oliveira Dias, Flavia Saldanha Kubrusly, Martha Massako Tanizaki, Elizabeth Angélica Leme Martins, Isaias Raw

**Affiliations:** 1 Instituto Butantan, São Paulo, SP, Brazil; 2 Fundação Butantan, São Paulo, SP, Brazil; Yale School of Public Health, United States of America

## Introduction

A recent review has discussed the economic value of vaccine for developed countries. The situation is quite different in developing countries, and we examine the situation in Brazil. Vaccines are of fundamental importance for the control of infectious diseases, especially among the population that lives in poor sanitary conditions. Also, vaccines can generate herd effects that result in protection even among those who have not been vaccinated, which can be of particular value to poor individuals who are not reached by health services. In appreciation of this importance, various international agencies, including the Pan American Health Organization (PAHO) and UNICEF, undertake large-scale procurement of vaccines for supply to developing countries. This scale of procurement has allowed these agencies to obtain very low prices. In Brazil, the Constitution includes the right to health care, which has led the government to formulate a goal of universal vaccination free of charge, a cost-effective measure against many important infectious diseases. Universal vaccination is a fundamental role of the federal, state, and municipal governments through the current unified public health care system (Sistema Único de Sáude [SUS]).

To achieve this goal, in 1985 the Ministry of Health (MH) launched a national immunization program and a plan to achieve self-sufficiency in vaccine production through local institutions. The latter program included support for innovation and technological development. The success of this platform has allowed the MH to purchase vaccines from domestic public vaccine production institutes at prices comparable to those obtained by PAHO and UNICEF. Most of the research institutes in less developed countries (LDCs) are the descendants of the various Pasteur-like institutes founded in the early 1900s. Throughout their lives, these institutions maintained scientific research programs but had limited capability for meeting good manufacturing practices (GMPs) in vaccine production. The implementation of the MH policy of local procurement required substantial investments to upgrade the production capabilities of these institutes.

The largest volume vaccine producer in Brazil is the Butantan Institute in São Paulo. The Institute, part of the São Paulo State Office of Health, was founded in 1901 to help in the control of bubonic fever and later became a producer of antivenoms and antitoxins. It maintains a scientific research program funded by grants from federal and state agencies. The administration of vaccine production is carried out by the Butantan Foundation, which is a separate, private, non-profit organization closely affiliated with the Institute (The Board of Directors of Butantan Institute and the Board of Curators of Butantan Foundation are composed of the same persons). Because of its legal structure, the Foundation is free of the usual administrative constraints of government agencies. In 2010, around 53 million doses of vaccine were used in the vaccination program implemented by the MH (Table 1). This comprises approximately 100 million doses of antigens, and of these, Butantan provided about 80%. The MH distributes vaccines free of charge to the whole country through about 25,000 health care centers, fulfilling a fundamental role of the SUS.

**Table 1:** Vaccination schedule in Brazil (vaccines provided by the Ministry of Health).

Vaccine	Age	Vaccine Doses Given in 2010^a^	Antigen Doses Produced in Brazil^b^	Antigen Doses Produced by Butantan	Producers
Intradermal BCG vaccine	At birth	3,121,271	3,121,271		Fundação Ataulpho Paiva, RJ, Brazil
Hepatitis B vaccine	At birth, 1 and 6 months	14,645,000	14,645,000	14,645,000	Instituto Butantan, SP, Brazil
Tetravalent vaccine (DTwP (diphtheria, tetanus, and pertussis) +Hib (*Haemophilus influenzae* b)	2, 4, and 6 months	8,550,731	34,202,924	25,652,193	DTwP – Instituto Butantan, SP, BrazilHib– GSK (technology transfer agreement with Bio-Manguinhos, RJ, Brazil)
OPV (oral polio vaccine)	2, 4, 6, and 15 months	41,771,039^c^	-	-	GSK (technology transfer agreement with Bio-Manguinhos, RJ, Brazil)
Rotavirus (monovalent oral human rotavirus vaccine)	2 and 4 months	5,125,267	-	-	GSK (technology transfer agreement with Bio-Manguinhos, RJ, Brazil)
Pneumococcal 10-valent conjugate vaccine^d^	2, 4, 6, and 10 months	6,747,277	-	-	GSK (technology transfer agreement with Bio-manguinhos/Fiocruz)
Meningitis C conjugate vaccine^d^	3, 5, and 15 months	4,104,357	-	-	Novartis (technology transfer agreement with Fundação Ezequiel Dias, MG, Brazil)
Yellow fever vaccine	9 months and booster every 10 years	6,699,459	6,699,459	-	Bio-manguinhos/Fiocruz, RJ, Brazil
MMR (measles, mumps, and rubella vaccine)	12 months and 4 years	5,856,491	-	-	GSK (technology transfer agreement with Bio-Manguinhos, RJ, Brazil)
DTwP (diphtheria, tetanus, and pertussis vaccine)	15 months and 4 years	5,456,881	16,370,643	16,370,643	Instituto Butantan, SP, Brazil
DT (diphtheria and tetanus vaccine)	Booster every 10 years	14,760,432	29,520,864	29,520,864	Instituto Butantan, SP, Brazil
Seasonal influenza vaccine	Once a year for those above 60 years of age	16,223,394	-		Sanofi-Pasteur (technology transfer agreement with Instituto Butantan)
Influenza H1N1 vaccine	Campaign in 2010	3,140,513	-		Sanofi-Pasteur (technology transfer agreement with Instituto Butantan), GSK, and Novartis
Pneumococcal 23-valent polysaccharide vaccine	Once for those above 60 years of age	249,773	-		Sanofi-Pasteur
Total		53,233,774	104,560,161	86,188,700	

a Source: Datasus (http://tabnet.datasus.gov.br/cgi/tabcgi.exe?pni/cnv/DPniuf.def), Ministry of Health (http://portal.saude.gov.br/portal/saude/profissional/area.cfm?id_area=1448), and Fiocruz (http://www.fiocruz.br/bio_eng/cgi/cgilua.exe/sys/start.htm?sid=208) and (http://www.fiocruz.br/bio_eng/cgi/cgilua.exe/sys/start.htm?infoid=549&sid=227).

b Considering the vaccines produced in Brazil (only those presenting all the steps in the production chain, such as BCG, Hepatitis B, DTwP+Hib, DTwP, DT and yellow fever), a total of 53,233,774 doses of vaccine were produced and given in Brazil. This represents 104,560,161 doses of antigens produced and given in Brazil. Butantan Institute produced 86.188.700 doses of these antigens (D, T, wP and hepatitis B).

c Includes vaccination campaigns and ^d^vaccines included in 2010 in the vaccination schedule.

d vaccines included in 2010 in the vaccination schedule.

The situation in Brazil contrasts with that of other developing countries. In India, private for-profit companies have emerged as the major vaccine producers. In China, the situation is mixed, with a number of government controlled non-profit vaccine production institutes and several emerging private sector producers. The private Indian manufacturers have, in several cases, become major vaccine exporters and sell large quantities to UNICEF. The Chinese manufacturers have largely remained as suppliers for local needs. In both India and China, major developed country for-profit vaccine manufacturers are buying interests in the local companies. There are no private sector for-profit vaccine manufacturers in Brazil.

## Vaccine Development

The Butantan Institute has employed four methods to obtain new vaccine technology.

1. Technology transfer from for-profit vaccine producers in developed countries
2. Technology transfer from public sector institutions in developed countries
3. Independent development
4. Partnerships with for-profit vaccine producers in less developed countries

## Technology Transfer from For-Profit Companies

While this approach can be successful, it has certain disadvantages. The companies are understandably reluctant to create competitors with knowledge and capabilities in the most up-to-date production methods and most advanced vaccines. Thus, the developing country partner may obtain out-of-date technology for older vaccines. Furthermore, the technology transfer agreements may not result in autonomous production capability, as they may require that the recipient of the technology obtains certain essential materials from the developed country supplier. The agreement may also impose a minimum price at which the vaccine can be sold, preventing the achievement of the most cost-effective programs. The agreement may not provide for the recipient partner to obtain new developments in production technology, imperiling sustained economic feasibility. If the agreement calls for stepwise technology transfer beginning with filling and labeling, there may be no guarantee of moving to the next step, resulting in a requirement to continue importing in bulk. The technology supplier may also demand quantities of vaccine for clinical trials without clear benefit for the developing country recipient or the country itself. Despite all the problems mentioned above, this model of technological transfer allowed a successful association between Butantan Institute and Sanofi-Pasteur for the production of seasonal influenza vaccine. The process started in 1999–2000 and the production of the first vaccine lots occurred in 2011. In the beginning, the vaccine was obtained ready for use and Butantan had to perform the quality control. This stage was followed by the implementation of the formulation and filling technologies, which allowed the purchase of the vaccine in bulk. In parallel, human resources were trained in all steps of the production chain and funds were obtained from federal and state agencies for the construction of a production plant. In 2008, the start up of the plant occurred. This important achievement is not only strategic for Brazil, but also for all of Latin America in the case of a pandemic influenza [1].

## Technology Transfer from Public Sector Institutions

As Butantan has proven itself to be technically competent with qualified personnel and modern facilities, it has sought new vaccine candidates from public health research institutes and universities in developed countries. In these programs, Butantan works in partnership to move from pilot-scale production to confirmation of proof of principle, to preparation of lots for clinical testing, and finally to large-scale production. Butantan is executing two such programs in collaboration with the United States National Institutes of Health for a pentavalent rotavirus vaccine and a tetravalent dengue vaccine. It is also undertaking programs with Children's Hospital Boston, of the Harvard Medical School, for a killed unencapsulated whole cell pneumococcal vaccine and with the Sabin Vaccine Institute and George Washington University for the *Necator* and *Schistosoma* parasite worms vaccines. Butantan is also working with the Infectious Diseases Research Institute in Seattle and the University of Washington on a *Leishmania* vaccine for dogs, which are the main reservoir for this disease in Latin America. The main advantage of this kind of partnership is that both sides will complement efforts to bring the potential vaccines to the market at a reduced time when compared with a development made by each partner alone. Depending on the case, the proof of principle in animal models was previously defined. Therefore, in these cases, the scale up of the bench process and the production under GMP conditions for the pre-clinical and Phase I/II clinical tests are the major challenges faced by the partners. It is important to point out that this kind of association is only feasible if the recipient institution counts with well trained human resources and is an active and qualified manufacturer with a well established market. From the point of view of the university and public research institutes involved, this represents a chance of high profit return and project success.

## Independent Development

**Figure 1:**
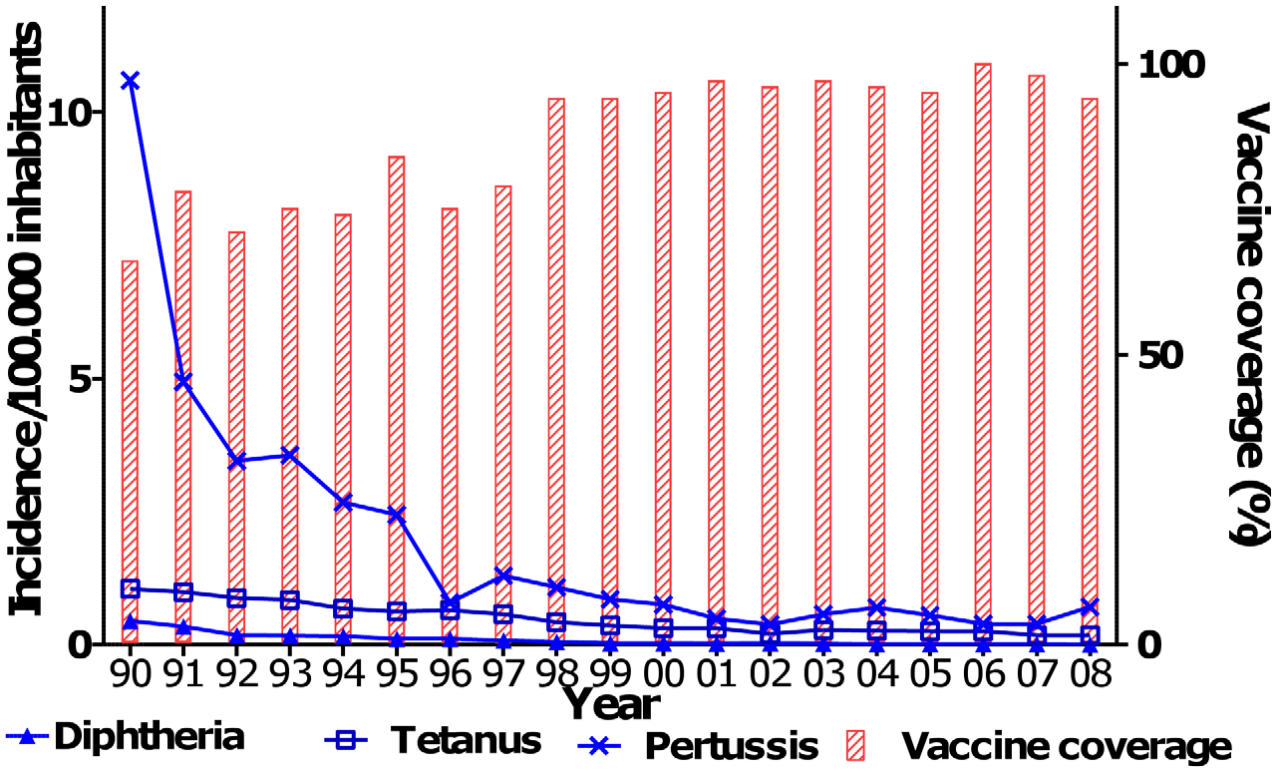
Coverage of DTP vaccination and incidence per 100,000 inhabitants of diphtheria, tetanus (except neonatal), and pertussis in Brazil from 1990 to 2008. Source: [12].

Since 1984, the Butantan Institute has produced diptheria, tetanus, and whole cell pertussis (DTwP) trivalent vaccine for the full cohort of children born each year in Brazil, which currently totals 3.2 million infants. This DTwP vaccine has been highly efficacious, lowering the incidence of all three diseases substantially between 1990 and 2008 (Figure 1). In contrast to the serious adverse reactions observed elsewhere, such events were not reported for the whole cell pertussis vaccine (wP) produced by Butantan. In Japan, the problem of adverse reactions has been solved by using isolated proteins in an acellular vaccine that has a production cost 50-fold higher than the whole cell vaccine [2]. The World Health Organization (WHO) and PATH have stated that there is no sound rationale for LDCs to replace whole cell vaccine with acellular vaccine. In Brazil, such replacement would increase the cost of vaccination against pertussis by about $100 million per year. To solve this problem, Butantan developed a process to remove lipopolysaccharide (LPS) from the bacterium to reduce inflammatory and febrile reactions. This new vaccine (wP_low_), formulated as DTwP_low_, can be supplied at the same price as the regular whole cell DTwP vaccine. This vaccine was subjected to the standard potency test of intra-cerebral challenge in mice and was shown to induce levels of protection similar to classical DTwP (W. Dias, A. van der Ark, M. Sakauchi, F. Kubrusly, A. Prestes et al., unpublished data). Furthermore, clinical evaluations were performed in infants and showed immunogenicity similar to classical DTwP and no significant side effects [3]. Production optimization of DTwP_low_ may increase the yield to about 200 million doses per year, allowing Butantan to export some quantities. Moreover, the LPS removed in this process can be hydrolyzed to monophosporyl lipid A (MPLA), a non-toxic product that can be used as an adjuvant for vaccines like those against influenza H5N1 and pandemic H1N1. The use of MPLA would allow a 4-fold reduction in antigen per dose, simultaneously increasing production capacity and lowering per-dose cost [4], [5]. As a byproduct of the production process of the wP_low_ vaccine, Butantan can produce kilograms of MPLA with only 10 micrograms needed per dose of influenza vaccine. Butantan has agreed to supply another Brazilian public sector vaccine producer, Fiocruz-Biomanguinhos, with DTwP_low_ ready for combination with lyophilized *Haemophilus influenzae* type b (Hib) vaccine. To date, Hib has been resuspended with DTwP by the staff at the health care centers to make the tetravalent vaccine right before use.

Since 1996, Butantan has produced recombinant hepatitis B vaccine, and 150 million doses have been administered to children and newborns. A tetravalent vaccine of DTwP_low_ and hepatitis B is under clinical trial and could be further formulated as a pentavalent vaccine by the addition of Hib produced by Fiocruz-Biomanguinhos. Regarding the Hib vaccine, its production by Fiocruz-Biomanguinhos is under a technology transfer agreement with GlaxoSmithKline that limits the export of the vaccine only to Mercosur countries (full members: Argentina, Brazil, Paraguay, and Uruguay; associate members: Bolivia, Chile, Colombia, Equador, Peru, and Venezuela). To overcome this limitation, Butantan is independently developing a Hib vaccine. This vaccine is expected to have a lower cost of production because of an innovative conjugation method for the polysaccharide antigen and the carrier protein resulting in higher yield. In addition, the carrier protein is also regularly produced by Butantan, avoiding its acquisition in bulk and resulting in a decrease of the final price. Thus, a pentavalent vaccine formulated with the Butantan Hib vaccine could perhaps be provided to LDCs at a price comparable to that obtained by PAHO and UNICEF. Butantan is particularly interested in multivalent vaccines because they have many advantages, such as inoculation of a single dose of adjuvant and the use of a single disposable syringe for many vaccines. In Brazil, the cost of vaccine administration is borne by municipalities and is not included by the MH in the cost of immunization. Between 2 and 18 months of age, each Brazilian receives seven injections to receive DTP, hepatitis B, and Hib vaccines (Table 1). Each dose contains aluminum hydroxide adjuvant, which, although used safely since 1926, is considered painful.

Butantan is also developing new pneumococcal vaccines that should be less expensive than existing ones. Pneumococcal-conjugated vaccines containing polysaccharide from seven, ten, or 13 serotypes have prices that are prohibitive for most LDCs. Also, these vaccines may induce serotype replacement, requiring development of new formulations containing additional serotypes [6]–[8]. Butantan is taking two approaches to develop pneumococcal vaccines. The most promising candidate was mentioned in the previous section and is a partnership with Children's Hospital Boston involving a *Streptococcus pneumoniae* strain without capsule as a simple whole cell inactivated bacterial vaccine. The second candidate employs the pneumococcal surface protein A (PspA). The combination of PspA with DTwP_low_ has been shown to improve protection against challenge with several pneumococcal strains in mice [9]. Furthermore, PspA may be conjugated with polysaccharides, reducing the need for a large number of polysaccharide serotypes in the vaccine.

A vaccine combining DTwP_low_, hepatitis B, Hib, and pneumococcal antigens could substantially reduce the cost to provide protection against a wide range of infections and with fewer injections. A further improvement would be the addition of the Salk inactivated polio vaccine. However, the development of such complex vaccines would require extensive clinical trials.

In Brazil, partial protection against tuberculosis is achieved by intradermal delivery of bacille Calmette-Gueri (BCG) at birth (Table 1). In addition, a first dose of hepatitis B vaccine at birth is being introduced to reduce mother-to-child transmission. A clinical trial has demonstrated the effectiveness and reduced pain of injection of a combined hepatitis B–BCG vaccine without aluminum hydroxide adjuvant in the first dose at birth, without changing the following second and third doses of hepatitis B vaccination (Table 1) [10].

**Figure 2:**
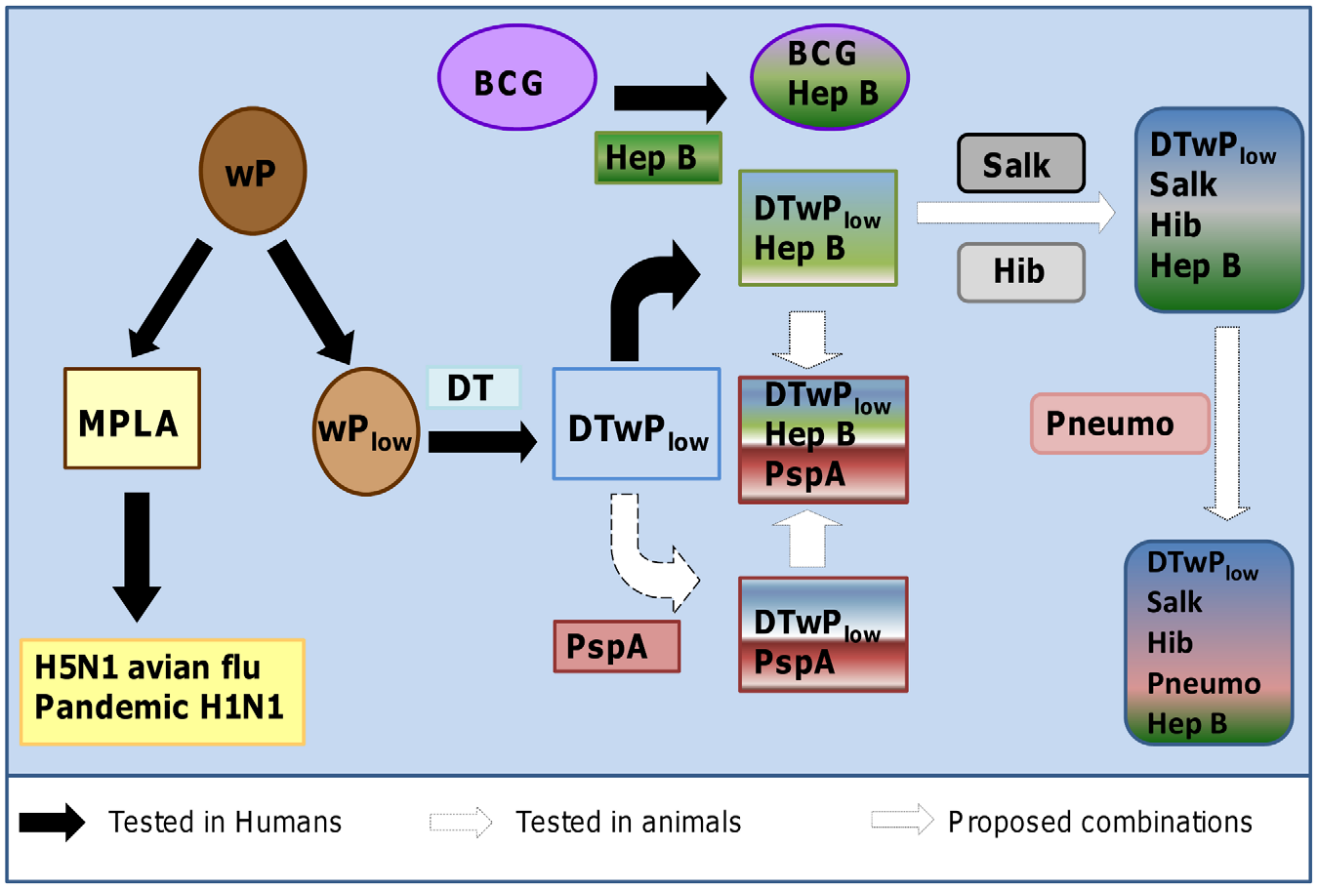
Schematic representation of combined vaccines as well as new vaccines under development at Instituto Butantan with the potential to impact public health.

The scheme in Figure 2 summarizes the planned development of new and combined vaccines at Butantan. These vaccines could provide substantial benefits for the national immunization program and thus for public health.

## Partnership with For-Profit Vaccine Producers in Less Developed Countries

With well trained personnel and a regular and qualified manufacturer, these features allowed Butantan to achieve the independent developments illustrated above. These are features also presented by other manufacturers in LDCs that may combine their efforts to develop new vaccines. In this case, for a successful partnership, a very clear agreement defining the fee, royalties, markets, and shares should be determined. Butantan is discussing this kind of partnership with members of the DCVMN (Developing Countries Vaccine Manufacturers Network) since DTwP_low_ can be a base for multiple vaccines (Figure 2) for LDCs. Such practice should be encouraged for all the members of the DCVMN for the benefit of all.

## Final Remarks

The national immunization program launched in 1985 in Brazil has provided immense public health benefits to the whole country free of charge to individuals. These benefits were achieved through sustained long-term efforts to develop all elements of the program, including product development, production, delivery, and disease surveillance. Vaccination of children, adults over 60 years, and persons with underlying health conditions that make them more susceptible to acquiring preventable infections with the existing vaccines provided by the MH and health care personnel are all covered free of charge through the SUS (Table 1). This is assured by the Brazilian Constitution and has created a large national market allowing sustainable vaccine production in contrast to the export-oriented market practiced by many private vaccine producers. Although this market is guaranteed by the government, there are several hurdles faced by Butantan. The production has to be done in advance, even when Butantan does not know the amount of vaccines to be purchased, which may vary from year to year. In addition, the payment depends on budget approval, which may also vary from months up to years. However, this experience, focusing on creation of domestic capabilities in the public sector to attend this demand, is positive and may present an interesting model for other LDCs not only to provide equitable health services, but also to develop sustainable technological institutions in vaccine development and production. Public vaccination resulted in the political decision of the MH to promote self-sufficiency in immunobiologicals, providing efficacious vaccines at an affordable cost and prioritizing vaccine production by a few public health Pasteur-like institutes that received support to renovate their production facilities and to introduce GMPs. This decision also led to other actions and necessities. To guarantee the quality of the vaccines, it created the independent National Immunological Control Laboratory (INCQS), which tests each lot of the vaccines delivered to the MH central storage facility (both located in Rio de Janeiro). If the lot does not conform with composition, potency, and safety requirements, it is destroyed and the producer is not refunded for its cost, incurring a hard penalty for non-compliance. This same analysis and certification are also applied for imported vaccines. Besides this, a federal regulatory agency (ANVISA) was created that certifies the production laboratories and promotes a move to full compliance with WHO GMP guidance, a process that is not instantaneous but depends on investments that the MH began to make available starting in 1985. All new plants, like Butantan's influenza plant, were built under these new regulations. Older plants are in a stepwise process of reform and if funds are available, total replacement will be considered. Funds for reform, replacement, or construction of a new plant are not an automatic process for Butantan, which belongs to the State of Sao Paulo. At this moment, Butantan is pursuing WHO prequalification in order to have permission to provide the vaccines to PAHO and UNICEF. Most of the production plants were built in the 1980s under past regulations and do not present the features necessary for current legislative approval. Therefore, modifications on the plants to conform to the new regulations are under way. It is important for a public producer that its vaccines be recognized as efficacious and safe as any other vaccine produced and prequalified by WHO in order to have public confidence and to become an important vaccine provider to other countries. Nevertheless, the vaccines that have been produced by Butantan since 1985 were approved by INCQS, the production plants were approved by ANVISA, and the massive immunization of 80 million children with about 350 million antigen doses in 26 years, as well as the immunization of 20 million adults over 60 years of age, was shown to be safe and efficacious, decreasing significantly the number of diseases (Figure 1). In order to continue with its mission to provide immunobiologicals for public health problems, Butantan needs to innovate constantly.

While collaboration between public sector institutions and private sector pharmaceutical companies (from both developed countries and LDCs) can also be successful and complementary for obtaining know-how for vaccine production, it is essential that the resulting products are affordable to ensure cost-effective programs. In addition, any adopted strategy must consider keeping high confidence in vaccines, which is usually greater among populations in LDCs than in industrialized countries [11]. Public confidence will allow uninterrupted delivery of immunization services and facilitate the introduction of new vaccines as they become available. Finally, the country should ensure the capability to undertake rigorous epidemiological studies to guide and evaluate the delivery of vaccination services.
